# Qualitative testing of EQ-HWB-9 self-complete and proxy versions in older adult-carer dyads in Hungary

**DOI:** 10.1186/s12955-026-02602-z

**Published:** 2026-08-11

**Authors:** Eszter Szlávicz, Ákos Szabó, Valentin Brodszky, Lea Lebar, Valentina Prevolnik Rupel, Kaja Zoran, Vesna Dolničar, Clara Mukuria, Fanni Rencz

**Affiliations:** 1 Department of Dermatology and Venereology, Medical School, University of Pécs, Akác utca 1, Pécs, HU-7632 Hungary; 2 Archiepiscopal College of Veszprém, Veszprém, Hungary; 3 Department of Financial Accounting, Corvinus University of Budapest, Budapest, Hungary; 4 Department of Health Policy, Corvinus University of Budapest, Budapest, Hungary; 5 Social Protection Institute of the Republic of Slovenia, Ljubljana, Slovenia; 6 Faculty of Social Sciences, University of Ljubljana, Ljubljana, Slovenia; 7 Institute for Economic Research, Ljubljana, Slovenia; 8 CM HE Consulting, Sheffield, England UK; 9 EuroQol Research Foundation, Rotterdam, The Netherlands

**Keywords:** Carer, Content validity, Dyad, EuroQol health and wellbeing, EQ-HWB-9, Modifications, Proxy, Qualitative

## Abstract

**Background:**

The EuroQol Health and Wellbeing 9 (EQ-HWB-9) is an experimental generic instrument designed for use in settings, populations and contexts where measuring wellbeing alongside health is relevant to decision making, such as in social care or in assessing impacts on carers. This study aims to assess the content validity of the EQ-HWB-9 self-complete and proxy versions in dyads of older adult social care users and their carers in Hungary, and to qualitatively compare the ‘experimental modified’ (2024) version with the ‘experimental’ (2022) version.

**Methods:**

In 2025, one-on-one, semi-structured interviews incorporating item-level probing and a card-sorting exercise were conducted with 15 dyads of older adult social care users and their carers in Hungary, using a convenience recruitment strategy. The self-complete version of EQ-HWB-9 was used for care recipients, while the proxy version (proxy-proxy perspective) was used for carers. We followed a standardised topic guide, focusing on three elements of content validity: comprehensibility, relevance and comprehensiveness. The interviews were analysed using framework analysis.

**Results:**

EQ-HWB-9 was positively viewed by both older adult care recipients and carers, with all items considered comprehensible and relevant by most respondents in the social care context. Carers’ interpretations of the proxy version aligned with those of care recipients. Three respondents considered the examples ‘working, shopping, housework’ in the day-to-day activities item not relevant. Ten respondents mentioned seven missing concepts, most commonly social relationships, community life and intellectual life. In terms of preferences for modifications, there was support for starting with day-to-day activities, using ‘difficulty’ rather than ‘trouble’ for the ‘concentrating/thinking clearly’ item, and ‘moderate’ over ‘some’ for the difficulty response scale. However, the experimental version’s wording was preferred for the mobility and control items. Overall, 28 of 30 respondents correctly ranked the cards in the card-sorting exercise for the modified frequency scale.

**Conclusions:**

This study confirmed the content validity of the experimental modified EQ-HWB-9 self-complete and proxy versions in Hungarian social care user older adult-carer dyads and provided systematic evidence on the proposed modifications to inform the finalisation of the instrument.

**Supplementary Information:**

The online version contains supplementary material available at https://doi.org/10.1186/s12955-026-02602-z.

## Background

As populations age in most developed countries, an increasing number of adults are living to older ages, placing a growing burden on health care and social care systems [1–4]. The key health and wellbeing areas affected by ageing include psychological problems, mobility difficulties, declining cognitive function, falls and other incidents, wounds and injuries, undernutrition and potential communication problems [5–8]. Some of these, particularly neurodegenerative diseases (e.g. dementia), general physical decline, balance problems and increased risk of falls, vision impairments, major chronic conditions (e.g. cardiovascular disease, stroke, cancer) and severe mobility problems (e.g. arthritis), may create a need for social care. Social care can help older adults maintain independence, support daily activities and promote social participation [9, 10]. Social care for older adults can be provided in different settings, most commonly through institutional care, such as residential care homes or nursing homes, or through home-based care delivered in the care recipient’s home. In both settings, care may be provided by paid formal professionals trained in areas such as nursing or social care. In home settings, however, informal care, typically provided on an unpaid basis. A growing proportion of the population provides informal care to family members, friends or neighbours, and the hours spent caring are also rising [11–13]. In Hungary, approximately 15–25% of the population acts as an informal carer for someone due to old age or other health problems or disabilities [14, 15]. Caring may have both negative (e.g. adverse physical and mental health effects, fatigue and risk of burnout) and positive experiences (e.g. life or compassion, satisfaction) for the health and wellbeing of carers [16–18].

Measuring outcomes is a key indicator of the efficient allocation of resources in both health care and social care. Given that caregiving has cross-sectoral effects, as it may reduce demand for health care [19, 20] and influences the health and wellbeing of both carers and care recipients, outcome measures that allow combined assessment using a standardised tool are particularly desirable. The EuroQol Health and Wellbeing (EQ-HWB) and its short version EQ-HWB-9 are instruments for use in settings, populations and contexts where measuring wellbeing in addition to health is relevant to decision making, for example in social care or in assessing impacts on carers [21, 22]. The EQ-HWB and EQ-HWB-9 are currently experimental instruments and remain under development. Several previous qualitative and quantitative studies have demonstrated the validity of the EQ-HWB(-9) in social care recipients and carers of children with chronic diseases and older adults, including patients with dementia in countries including the US, Australia and China [23–29]. Few studies have also provided evidence on the validity of the proxy version [30, 31]. A proxy version is a version of the instrument completed by a respondent on behalf of another person, typically when that person is unable or less able to self-report [32]. This is particularly relevant in social care contexts, where carers often act as proxies [33].

Since the release of the experimental (v1.1; 2022) version of EQ-HWB-9, the developer team has proposed several minor modifications to wording, item order, response levels and layout (i.e. item stems), resulting in the ‘experimental modified’ version (v1.2; 2024) (Appendix 1). These modifications require qualitative and quantitative testing to support decisions on which changes should be accepted or rejected. Such testing is required across multiple populations, languages and contexts, and should include both self-completed and proxy-reported assessments, given the future intended use of the instrument.

This study aims to qualitatively assess the content validity, including comprehensibility, relevance and comprehensiveness, of the modified EQ-HWB-9 self-complete and proxy versions in dyads of older adult social care users and their carers in Hungary. It forms part of a series of qualitative evaluations of EQ-HWB-9 modifications across multiple countries and populations, comparing the modifications with the experimental (2022) version to contribute to the evidence base that the developer team will use to finalise the instrument.

## Methods

### Study design

We conducted a cross-sectional study using one-on-one, semi-structured interviews between May and August 2025 with dyads of older adult social care users and their carers in Hungary, following a standardised topic guide. All participants were interviewed in person. We aimed to conduct the interviews with the care recipient and carer on the same day or not more than 3 days apart. Written informed consent was obtained from each participant. All interviews were conducted by the first author, a medical doctor and licensed psychotherapist with a PhD in clinical medicine, who has previous experience in qualitative research using EuroQol instruments and in interviewing vulnerable populations. The language of the interviews was Hungarian. Data quality of the interviews was monitored by the last author, and the interviews were regularly discussed among the study team members. Reporting follows the Consolidated criteria for reporting qualitative research (COREQ) checklist [34].

### Study population

A convenience recruitment strategy was applied to recruit 15 older adult social care user and carer dyads in Hungary, where the care recipient was older than 65 years. The sample size was determined in advance based on feasibility, the minimum sample size required to gather key information based on previous qualitative work with EuroQol instruments [30, 35] and the timelines of the EQ-HWB-9 development programme. The study aimed to obtain sufficient depth of information to address the research objectives rather than to achieve thematic saturation. Participants were recruited from three nursing homes located in three Hungarian cities (Hévíz and Sümeg in Western Hungary and Pécs in Southern Hungary) and through the first author’s professional networks. Participants were eligible for inclusion if they had lived in Hungary within the past 10 years, were fluent in Hungarian, provided or received formal or informal care for at least 4 h in an average week, had the cognitive ability to read and interpret questions and provided written informed consent. The threshold of ‘at least 4 hours per week’ was used to ensure that carers had regular caregiving involvement sufficient to meaningfully complete proxy assessments and speak about the care recipient, while also avoiding an unnecessarily high threshold that would exclude potential participants. In addition, care recipients were required to be aged 65 years or older, while carers were required to be aged 18 years or older. The age of 65 was defined as it was the retirement age at the time of the study and is used by the Hungarian Central Statistical Office to define older age in Hungary [36].

### EQ-HWB-9

The EQ-HWB-9 is a generic instrument to measure health and wellbeing, consisting of nine items: day-to-day activities, getting around inside or outside, exhaustion, loneliness, concentrating/thinking clearly, anxiety, sadness/depression, control and physical pain [21]. The recall period is the last 7 days, and each item uses a 5-point response scale measuring either difficulty, frequency or severity (Appendix 1). EQ-HWB-9 v1.1 (experimental, 2022) has previously been validated across multiple countries and studies in the general population, patients and carers [27, 28, 37–43].

### Translation of EQ-HWB to Hungarian

The translation work was a preliminary step that preceded and enabled the main study, rather than forming part of the study methodology itself. As no Hungarian translation of the EQ-HWB (short or long form) existed, the study team conducted translation and cognitive debriefing following the EuroQol Version Management Committee (VMC) guidelines. Two independent Hungarian translators performed forward translations of the long form, followed by the creation of a consensus version and VMC review. Two native English-speaking translators then performed backward translations, leading to a second consensus version reviewed by the VMC. The self-complete, interviewer-administered and proxy versions (proxy-proxy perspective) were translated for both the experimental and experimental modified (hereafter: modified) versions. Cognitive debriefing occurred in two steps. First, eight respondents (mix of patients and healthy adults) were asked about the experimental long-form self-complete version, were probed on the modifications, and then asked about the experimental interviewer-administered version’s instructions. Second, another eight respondents were asked about the modified EQ-HWB long form, with differences from the experimental version probed and proxy instructions assessed at the end. All cognitive debriefing interviews were conducted by the first author. A third consensus version was created, reviewed by the VMC, proofread by the research team, and finalized as the Hungarian EQ-HWB versions. All experimental and modified EQ-HWB-9 versions (self-complete, interviewer-administered and proxy) were extracted from their respective long forms. The translation process, including forward and backward translation and 16 cognitive debriefing interviews, was conducted between March 2024 and March 2025.

### Interview process

Although all available EQ-HWB versions were translated, this study focused on testing only the self-complete (in care recipients) and proxy (in carers) EQ-HWB-9 versions with particular emphasis on exploring perceived differences between the experimental and modified versions. The proposed EQ-HWB-9 modifications tested included wording changes to the mobility, concentration/thinking clearly and control items, layout (i.e. removing stems) and reordering the mobility and day-to-day activities items, and changes to one response level in both the difficulty and frequency scales. We followed the standardised topic guide developed by EuroQol’s EQ-HWB Working Group for qualitative testing of EQ-HWB modifications based on the COSMIN (Consensus-based Standards for the selection of health Measurement Instruments) guidelines [44]. We focused on three elements of content validity: comprehensibility, relevance and comprehensiveness. The procedures described below focus on EQ-HWB-9; additional long-form items and potential EQ-HWB visual analogue scale (VAS) versions were included for exploratory purposes and will be reported elsewhere.

During the interviews, first, as a warm-up task and to familiarize respondents with the context of the interview, participants were asked about their views on the meaning and important aspects of health, health-related quality of life, wellbeing and their experience with health and social care. After this, respondents completed a card-sorting exercise in which they were asked to order the modified frequency response options (‘none of the time’, ‘a little of the time’, ‘sometimes’, ‘often’, ‘most or all of the time’). The aim of the exercise was to check understanding of the response options and confirm whether respondents ordered them correctly. Cards were presented to respondents in a random order. Participants were asked to look at the words on the cards and then arrange them in the order they felt was appropriate, with the option to place two or more cards next to each other if they considered them equivalent. Next, they reviewed each item in the modified EQ-HWB-9 together with the interviewer – without completing it – while responding to targeted probing questions about the comprehension and relevance of each item. After reviewing all nine items, respondents were asked about the comprehensiveness of the overall questionnaire, particularly whether any important key concept was missing or if any items were redundant. Then, respondents were presented with the experimental EQ-HWB-9, placed on the table next to the modified EQ-HWB-9 so that they could see both versions. They reviewed the two together while the interviewer asked specific probing questions about each modification, including comprehensibility and preference for the experimental or modified version. After this, a few selected questions from the EQ-HWB long form were presented to respondents (both modified and experimental), particularly those that had undergone major changes, and were probed. This was followed by a review of a couple of potential EQ-HWB VAS versions. Further details and the results of this additional assessment will be reported elsewhere.

At the end of the interview, each respondent was asked to complete the EQ-HWB-9, along with the selected additional EQ-HWB long-form items and the EQ-HWB VAS versions used in the interview. This was followed by a very brief background questionnaire asking about their socio-demographic background (e.g. age, gender, level of education, place of living (urban vs. rural), health (e.g. history of chronic diseases, disability) and social-care related information (e.g. type of care (formal or informal, institution- or home-based), average weekly hours of care received or provided and the relationship between the carer and care recipient).

### Data analysis

All interviews were audio-recorded and transcribed verbatim in Hungarian. Transcripts were analysed by study team members speaking the language, using framework analysis [45]. The analytical framework developed by EuroQol’s EQ-HWB Working Group was followed, aligning with the COSMIN content validity testing guidance [44]. As part of this process, a customized Microsoft Excel extraction file was used. Each item was coded for: (i) understanding (yes, partial, no); (ii) relevance (yes, no); (iii) preferences between modified and experimental items, including the reasons for these preferences (experimental preferred, modified preferred, no preference, no difference).

We categorised understanding issues with the items into six groups. U1 refers to wrong or ambiguous interpretation, where respondents understood the item differently from the developers’ intent. U2 reflects difficulty interpreting or odd wording, arising from unfamiliar terms or awkward phrasing. U3 indicates a narrow interpretation, with respondents focusing on only part of the construct. U4 concerns translation mismatches, where the word used does not convey the intended meaning. U5 arises when items include multiple aspects, making it difficult for respondents to provide a single answer. U6 relates to issues, where the examples provided led to misinterpretation of the item. Partial misinterpretation means that the person fundamentally understands the question, but their interpretation is not entirely correct. Despite the partial misinterpretation, the examples can still be appropriate.

All interviews were coded by the first author, and the coding was reviewed by the last author, who regularly met with the first author to discuss any disagreements and reach consensus. Verbatim characteristic quotes supporting each category were also extracted and translated to English. We report all results separately for carers (CA) and care recipients (CR), as well as in combined analyses.

## Results

### Characteristics of the study population

Seventeen dyads were invited to participate; however, in two cases, the carer declined participation, and the dyad was therefore not interviewed. Both dyads that declined participation were in the informal care context. Overall, 15 older adult carer dyads completed the interviews. In one instance, a care recipient residing in a nursing home was identified as being 63 years old only after the interview based on the background questionnaire. The research team decided not to exclude this participant and their carer from the study. In one dyad, there were more than three days (7 days) between the interviews of the care recipient and the carer. Since there were no significant fluctuations in the care recipient’s condition, we retained the data for this dyad. The average interview duration was 45 min for care recipients (range: 21–75 min) and 35 min for carers (range: 18–57 min). Although some participants reported visual impairments, all respondents were able to read the questionnaire independently.

Thirteen of the 15 included care recipients lived in nursing homes, and two care recipients received home care from formal paid carers. All caregivers were professional formal carers, and therefore no family or other personal relationships existed between the carers and care recipients. The median age of care recipients was 79 years (range 63–91), with female predominance (Table 1). They typically had secondary or tertiary education (*n* = 11), and most of them were widowed, divorced or single (*n* = 13). All care recipients had at least one chronic condition, with the most common being hypertension, cardiovascular disease, visual impairments and arthritis. In comparison, all carers were female, and their median age was 47 years (range 23–68). Most carers had a secondary level of education (*n* = 12) and approximately half of them were married or living with a partner (*n* = 8). The hours of care provided for the care recipient in the study ranged from 4 to 12 h.

**Table 1:** Characteristics of the study population

		Care recipient (*n* = 15)	Carer (*n* = 15)
Age (median, range years)		79 (63–91)	47 (23–68)
Gender	Female	11 (73%)	15 (100%)
	Male	4 (27%)	0 (0%)
Education	Primary	4 (27%)	2 (13%)
	Secondary	7 (47%)	12 (80%)
	College/university	4 (27%)	1 (7%)
Marital status	Divorced	5 (33%)	1 (7%)
	Married	2 (13%)	4 (27%)
	Partnered	0 (0%)	4 (27%)
	Single	2 (13%)	5 (33%)
	Widow	6 (40%)	1 (7%)
Median (range) weekly hours of care received/provided		6 (4–12)*	
Institutionalised care		13 (87%) dyads	
Home care (formal)		2 (13%) dyads	
Chronic conditions**			
3 chronic conditions		3 (20%)	-
≥ 3 chronic conditions		12 (80%)	

* In the case of nursing home residents, it was possible that more than one carer provided care to the care recipient. We report the number of hours received from the carer who was interviewed. **No care recipients had 0, 1 or 2 chronic conditions. The most prevalent chronic conditions in the sample were hypertension (*n* = 12), cardiovascular disease (*n* = 9), visual impairment (*n* = 9), arthritis (*n* = 8), cerebrovascular disease (*n* = 6), gastrointestinal disease (*n* = 5) and urinary incontinence (*n* = 5)

### Card sorting exercise

The aim of the card sorting exercise was to assess understanding of the frequency response options and to confirm whether respondents ordered them correctly. The vast majority of the participants (28 of 30) ranked the cards in the expected order, with the exception of one carer and one care recipient. These participants reported that ‘sometimes’ indicated a more frequent event, than ‘a little of the time’ (woman, 73, CR) or ‘a little of the time’ and ‘sometimes’ conveyed the same meaning, similarly to ‘most or all of the time’ and ‘often’ (woman, 43, CA). Notably, the care recipient who misordered the response options expressed difficulties in understanding the card sorting task. Overall, these findings suggest that the modified frequency response options were well understood by participants, with only isolated instances of misinterpretation.

### Comprehensibility of EQ-HWB-9

In general, participants interpreted the individual questionnaire items as intended. Problems of understanding were identified only in few cases and often were partial in nature (Table 2). No problems of understanding were reported for three items: ‘day-to-day activities’, ‘lonely’ and ‘concentrating/thinking clearly’. The most common misinterpretation concerned ‘depression’, which some respondents equated with anxiety (n = 3). One carer stated: “*Depression is when someone is constantly anxious”* (man, 71, CR). Similar interpretations were expressed by two care recipients: “*Being depressed is being nervous about something”* (woman, 91, CR) and “*Depression means to be nervous and all that*” (man, 81, CR). One of these participants already indicated in response to the preceding ‘anxiety’ item that she considered depression to be synonymous with anxiety: *“I would call anxiety depression. Anxiety at work is a good example. Someone is always afraid of losing their job.”* (woman, 91, CR). However, her quote suggests that despite the partial misinterpretation, she provided relevant examples for ‘anxiety’.

**Table 2:** Understanding and relevance of the EQ-HWB-9

Questionnaire item		Total			Care recipient			Carer		
		Yes	No	Partial	Yes	No	Partial	Yes	No	Partial
Day-to-day activities	**Understanding**	30	0	0	15	0	0	15	0	0
	**Relevance**	29	1	0	14	1	0	15	0	0
Getting around inside or outside	**Understanding**	28	1 (U1)	1 (U4)	13	1 (U1)	1 (U4)	15	0	0
	**Relevance**	30	0	0	15	0	0	15	0	0
Exhausted	**Understanding**	29	1 (U1)	0	14	1 (U1)	0	15	0	0
	**Relevance**	28	1	1	13	1	1	15	0	0
Lonely	**Understanding**	30	0	0	15	0	0	15	0	0
	**Relevance**	29	1	0	14	1	0	15	0	0
Concentrating/thinking clearly	**Understanding**	30	0	0	15	0	0	15	0	0
	**Relevance**	30	0	0	15	0	0	15	0	0
Anxious	**Understanding**	29	0	1 (U1)	14	0	1 (U1)	15	0	0
	**Relevance**	30	0	0	15	0	0	15	0	0
Sad/depressed	**Understanding**	27	0	3 (U1)	13	0	2 (U1)	14	0	1 (U1)
	**Relevance**	30	0	0	15	0	0	15	0	0
Control	**Understanding**	29	1 (U3)	0	14	1 (U3)	0	15	0	0
	**Relevance**	30	0	0	15	0	0	15	0	0
Physical pain	**Understanding**	29	0	1 (U1)	14	0	1 (U3)	15	0	0
	**Relevance**	30	0	0	15	0	0	15	0	0

U1 = wrong or ambiguous interpretation, where respondents understood the item differently from the developers’ intent; U3 = a narrow interpretation, with respondents focusing on only part of the construct; U4 = translation mismatches, where the word used does not convey the intended meaning

For the remaining items ‘getting around inside or outside’, ‘exhaustion’, ‘anxiety’, ‘control’ and ‘physical pain’, only sporadic errors of interpretation were identified, affecting one or two respondents. Importantly, for the ‘getting around inside or outside’ item, the reference to aids in the UK English source version *(‘using*,* for example*,* walking stick*,* frame or wheelchair*,* if you usually use them’)* caused problems of understanding during the cognitive debriefing as part of the translation. It was therefore rephrased as *‘If you use*,* for example*,* a walking stick or wheelchair*,* please consider their use when answering.’* This revised version was used and well understood by all 30 respondents in the present study. Only one misinterpretation related to this item was identified: *“I don’t understand this … She definitely needs to use the wheelchair.”* (woman, CA, 43). One care recipient interpreted ‘getting around inside or outside’ as referring to transportation rather than mobility: “*I also drive a car. At first*,* because of the stoma bag*,* I said I’d end my life here*,* because I can’t live like this (…) But I’ve also met other people who have stomas and drive tractors and do agricultural work.*” (man, 76, CR). For the ‘control’ item, one participant living in a nursing home commented: *“I don’t really understand this question. What influence can I have on everyday life here?”* (woman, 70, CR). For ‘physical pain’, one participant interpreted ‘pain’ as physical or verbal abuse (woman, 70, CR).

### Relevance of EQ-HWB-9

Overall, the EQ-HWB-9 was considered relevant in the social care context in Hungary. Getting around inside or outside and the last five items (concentrating/thinking clearly, anxiety, sadness/depression, control and physical pain) were deemed relevant by all 30 respondents (Table 2). The remaining three items were not considered relevant by one or two respondents each. For the day-to-day activities item, a care recipient living in a nursing home questioned its relevance: *“Housework is not our job. They do the bed linen. I go down for food in the morning and at noon. They bring dinner. I don’t know if this applies to me*,* do I do it or does someone else do it for me? (…) I can do very little*,* I don’t know what to say.”* (woman, 73, CR). Similar concerns were raised for the ‘exhaustion’ item in the nursing home context: “*This is not a good question*,* since a person has nothing to do*,* so there is nothing to get tired of”* (man, 69, CR). Three CRs made suggestions to better adapt the questions for nursing home residents, such as adding *‘if you usually do this at all*’ to the end of the day-to-day activities item, or changing the current examples of *‘working*,* shopping*,* housework’* to *‘dressing and hygiene’*.

### Comprehensiveness of EQ-HWB-9

The majority of respondents (10 CAs and 10 CRs) rated the questionnaire as comprehensive and did not report any missing concepts. There were generally positive opinions about the questionnaire, both from the aspects of carers and care recipients. Carers stated, for example: *“It fully presents the physical and mental problems of the elderly.”* (woman, 47, CA); “*The questions are simple*,* but the answers go deep. (…) It had everything that is important for an elderly person. (…) It really has everything*,* company*,* necessities.”* (woman, 40, CA) and “*It was understandable*,* interesting*,* and there is definitely a need for something like this. (…) I think it was quite comprehensive.*” (woman, 40, CA). Opinions from care recipients were similar: “*The questionnaire is very useful for people in this mental state who are cared for here. It sheds light on the conditions very well. I recognized myself in many ways. I think it’s good that way. (…) “It contains all the things that an elderly person experiences.”* (man, 76, CR); “*The questionnaire was important*,* everyone should know about these things and talk about them. (…) I don’t see anything missing in it.”* (woman, 82, CR) and “*Very good. This is really a questionnaire for us.”* (woman, 91, CR).

The remaining 10 participants (5 CAs and 5 CRs) reported overall 7 missing concepts (Table 3). Examples include the following: social relationships (e.g. interpersonal contacts, close relationships and talking to others; n = 8), community engagement (n = 4) and cognitive engagement (n = 2). One respondent commented on how she disliked the negative framing of the psychosocial items: *“These are all such negative questions. Exhaustion*,* loneliness*,* difficulty concentrating*,* depression*,* sadness. (…) There are no positive questions in it that would highlight the positivity*.” (woman, 53, CA).

**Table 3:** Comprehensiveness and redundancy of the EQ-HWB-9

		Example quote	Total	Care recipient	Carer
**Comprehensiveness**	**No missing aspects**	-	20	10	10
	Missing aspects*	-	10	5	5
	Social relationships*	-	8	4	4
	*Interpersonal contacts (i.e. broad*,* general contact with others)*	“The contact with others where I am, with the people around me.” (…) “What it is like with those who are around me.” (woman, 85, CR)	3	1	2
	*Close relationships (with family*,* carers*,* emotionally significant ties)*	“Or perhaps regarding their family, what their relationship with them is like. Or even with us, the carers. What they feel in relation to us?” (woman, 53. CA)	3	2	1
	*Talking to others (i.e. communication with others as an activity/need)*	“Having an item about conversation would be good.” (woman, 73, CR)	3	2	1
	Community engagement	“I would also ask how they experience community life.” (woman, 76, CR)	4	2	2
	Cognitive engagement (i.e. mental stimulation and interest)	“It is not enough to describe the resident. It is true that there are questions here about feeling sad or anxious, which could also be mental, so psychological aspects, what interests them, or what used to interest them.” (woman, 53, CA)	2	1	1
	Physical condition	“There are many other things besides this. For example, a person’s physical condition.” (woman, 73, CR)	1	1	0
	Psychological wellbeing (mental and emotional state)	“Or their mental state, mind and soul. These are things they can only deal with themselves, or it would require a much deeper level of engagement.” (woman, 73, CR)	1	1	0
	Fear of death	“I thought there would be a question about whether we are afraid of death.” (woman, 76, CR)	1	1	0
	Lived experience of the place of residence	“And the place where they live, although it does include feeling lonely, it could be expanded to how they experience everyday life there.” (woman, 53, CA)	1	0	1
**Redundancy**	**No redundancy**	-	28	14	14
	**Redundancy**	-	2	1	1
	*Anxiety and sadness/depression*	“Items 6 [anxiety] and 7 [sadness/depression]” (man, 71, CR)	1	1	0
	*Loneliness and anxiety; loneliness*,* exhaustion and sadness/depression*	“Item 4 [loneliness] and item 6 [anxiety] can be merged. And item 3 [exhaustion] can be combined with item 7 [sadness/depression]. Because depression is a kind of mental fatigue. It also includes loneliness.” (woman, 43, CA)	1	0	1

Note: Some quotes reflect suggested items rather than direct conceptual definitions

* Some participants mentioned more than one missing concept, and more than one interpersonal-relationship-related missing concept may have been identified by a single respondent

Regarding redundancy, most respondents (*n* = 28) perceived the items as distinct. However, one participant highlighted the redundancy of items ‘anxiety’ and ‘sadness/depression’ (man, 71, CR). A carer suggested merging multiple items, due to redundancy: *“Item 4 (loneliness) and Item 6 (anxiety) can be merged. And Item 3 (exhaustion) can be combined with item 7 (sadness/depression). Because depression is a kind of mental fatigue. It also includes loneliness.”* (woman, 43, CA). Conversely, another participant stated, that despite the similarities, the questionnaire should be kept in its current form: “*I’ve talked about the same thing several times. About exhaustion*,* and loneliness*,* about how it affects everyday life. But I’d like to keep it as a separate issue.*” (woman, 82, CR).

### Preferences between modifications

Of the seven modifications tested, both care recipients and carers more frequently preferred the modified over the original version in four cases: the day-to-day activities first item; changing ‘trouble’ to ‘difficulty’ for the concentrating/thinking clearly item; using ‘moderate’ instead of ‘some’ for the difficulty scale; and removing item stems (Table 4). For the mobility item, the conjunction ‘and’ was preferred because of its broader scope (n = 16 vs n = 3): “*And. From my perspective*,* I walk both indoors and outdoors.*” (man, 76, CR). However, 10 respondents did not express any preference. Considering the order of the items, starting with day-to-day activities was preferred by the majority of respondents (n = 19), for example: “*Daily activities begin with getting up at home or wherever we are. And only then do we start moving around*” (woman, 51, CA). For the concentrating/thinking clearly item, ‘difficulty’ was preferred over ‘trouble’ (n = 22 vs. n = 4). An important remark related to the modification of the ‘control’ item is that in Hungarian, ‘lack of control’ has the same meaning as ‘no control’, therefore only the difference between the part in parentheses could be tested. Eighteen participants preferred the experimental, longer version in parentheses, however, the difference was small, and respondents also liked the modified version: *“It’s the same in content*,* but the other one is worded longer. However*,* the message of the question is the same.”* (woman, 46, CA). For the difficulty scale, ‘moderate difficulty’ was preferred over ‘some difficulty’ (n = 20 vs. n = 9), for example: “*The moderate one*,* because it represents a half-and-half ratio*” (woman, 43, CA). For the frequency scale, ‘only occasionally’ was preferred over ‘a little of the time’ (n = 17 vs. n = 9). There was also a general preference for removing the item stem (n = 18 vs. n = 12). Regarding this last finding, a participant stated that: “*I read it once and understood it like this. I don’t think it needs to be repeated all the time.”* (woman, 51, CA). Participants were very often unable to provide a reason for their choice and tended to decide based on which option sounded better to them.

**Table 4:** Comparison of the experimental modified and experimental EQ-HWB-9

		Experimental modified (EM)	Experimental (E)	No preference	No difference	Missing
Mobility item conjunction: “or” (EM) vs. “and” (E)	Total	7	19	2	2	0
	CR	5	8	1	1	0
	CA	2	11	1	1	0
Item order: day-to-day activities first, mobility second (EM) vs. mobility first, day-to-day-activities second (E)	Total	16	3	10	0	1
	CR	6	3	5	0	1
	CA	10	0	5	0	0
Concentrating/thinking clearly: “difficulty” (EM) vs. “trouble” (E)	Total	22	4	2	2	0
	CR	9	4	1	1	0
	CA	13	0	1	1	0
Control*:*“had no choice to do things or have things done for you as you liked and when you wanted”* (E) vs. *you had no choice over what you did or how things were done for you* (EM)	Total	12	18	0	0	0
	CR	6	9	0	0	0
	CA	6	9	0	0	0
Difficulty scale: “moderate” (EM) vs. “some” (E)	Total	20	9	0	1	0
	CR	10	5	0	0	0
	CA	10	4	0	1	0
Frequency scale: “a little of the time” (EM) vs. “only occasionally” (E)	Total	9	17	2	1	1
	CR	4	7	2	1	1
	CA	5	10	0	0	0
Removed item stems (EM) vs. item stems (E)	Total	18	12	0	0	0
	CR	8	7	0	0	0
	CA	10	5	0	0	0

*In Hungarian, ‘lack of control’ has the same meaning as ‘no control’, so only the difference between the part in parentheses could be tested. CA = Carers; CR = Care recipient; E = experimental EQ-HWB-9 (2022); EM = experimental modified EQ-HWB-9 (2024)

## Discussion

This study qualitatively validated the Hungarian versions of the self-complete and proxy EQ-HWB-9 modified versions among older adult care recipients and carer dyads. In our study, all EQ-HWB-9 items were considered relevant by most respondents in the social care context, providing valuable validation evidence for one of the intended areas of use of the EQ-HWB-9, particularly as all previous qualitative validation studies in the aged care context have focused on only one of these versions rather than both [30]. Our study is the first to provide qualitative evidence on the proxy version of the modified EQ-HWB-9 in the older adult social care context. Overall, the modified EQ-HWB-9 was positively viewed by both older adult care recipients and their carers in Hungary, and no major problems were reported in terms of understanding, relevance or comprehensiveness in this context. Carers’ interpretations of the proxy version were broadly aligned with care recipients’ self-reported interpretations. The slight wording change in the parentheses of the mobility item, compared with the UK source version (*‘If you use*,* for example*,* a walking stick or wheelchair*,* please consider their use when answering.’)*, appeared to work very well in Hungarian, improving understanding that had previously caused confusion for some respondents in other studies, who were sometimes unsure whether the questionnaire was asking about the use of mobility aids [26, 30]. Some findings confirm those reported in two previous studies in Australia in the aged care context, such as the irrelevance of examples in the day-to-day activities item [30, 46].

The ‘loneliness’ item in the EQ-HWB-9 is intended to cover the domain of social relationships [21] and was considered understandable by all respondents and relevant by all but one. However, seven respondents reported that social relationships (e.g. interpersonal relationships, close contacts, talking to others) were missing from the questionnaire. While other aspects of social connections besides loneliness were considered as part of the development of the EQ-HWB (e.g. belonging, positive relationships, stigma, support), they were regarded as related, and loneliness received the most support during the consultation process [21]. The EQ-HWB-9 is a generic measure intended for use across multiple populations, including social care users, carers (also for self-assessment) and long-term patients. Therefore, the absence of a single concept identified in only one country or in only older adult social care users and their carers may not, on its own, justify revisiting previous decisions in the instrument’s development. Furthermore, given that the EQ-HWB-9 may be used for preference elicitation and the development of value sets, its items need to be independent. Therefore, the addition of a social relationships item would likely violate this principle, at least partially. Other missing concepts were also identified by a small number of participants, including community engagement, cognitive engagement, psychological wellbeing (mental or emotional state) or fear of death. As with other generic measures, EQ-HWB-9 is not intended to capture all aspects of health and wellbeing, and qualitative interviews will inevitably elicit additional concepts reflecting individual experiences. These findings are therefore primarily informative for scientific understanding within this sample and do not necessarily indicate a need for immediate changes to the descriptive system.

There are two different proxy perspectives that can be used for health and wellbeing measures: proxy-proxy and proxy-person [32]. This study used the proxy-proxy perspective in carers, which asks how the proxy would rate the care recipient’s status, whereas the proxy-person perspective focuses on how the proxy believes the care recipient would rate their own status. These two approaches can produce different results because they reflect different cognitive tasks, leading to two types of discrepancy: the inter-rater gap (between care recipient and proxy-person ratings) and the intra-proxy gap (between proxy-person and proxy-proxy ratings) [32]. While differences between these perspectives have been widely studied using other measures (e.g. QOL-AD, EQ-5D), limited evidence is available for EQ-HWB-9 [47]. In our study, there were no specific problems with adhering to the proxy-proxy perspective, consistent with a previous qualitative EQ-HWB-9 study in Germany [48]; however, another study from Australia suggested that carers may sometimes mix perspectives and respond in a proxy-person manner [49]. These previous studies have also reported that carers find items with feeling-framed wording or symptom framing (e.g. anxiety, sadness/depression and pain items) more challenging to complete when using a proxy-proxy perspective [48, 49]. Future research is needed to further examine the validity of different proxy perspectives for EQ-HWB-9 using both qualitative and quantitative approaches.

When respondents were shown the previous item and response scale versions (from the experimental 2022 version) preferences were expressed; however, few respondents provided clear reasoning for these preferences for most modifications. In general, starting the questionnaire with ‘day-to-day activities’ was supported, as was using ‘difficulty’ rather than ‘trouble’ for the concentration/thinking item and ‘moderate’ over ‘some‘ for the difficulty response scale. For most items, however, the primary justification was that one option simply ‘sounds better’ which cannot be considered a strong argument beyond natural language preference. In some cases, the experimental version was favoured upon probing; for example, ‘and’ over ‘or’ for mobility or ‘only occasionally’ over ‘a little of the time’ for the frequency scale. This does not imply that the modified version is inferior, as it was still viewed positively overall and demonstrated good comprehensibility. A good example of this is that, while ‘only occasionally’ was preferred by many respondents over ‘a little of the time’ by more than half of the respondents; however, 28 of 30 participants correctly ranked the cards in the card-sorting exercise for the modified frequency scale featuring the response option ‘a little of the time’. Our findings contribute to an international programme of work on the qualitative and quantitative testing of EQ-HWB-9 modifications. Alongside other countries (Argentina, Australia, China, Germany, Indonesia, Ireland, the Netherlands, Slovenia, the UK and the US), this study provides evidence on the validity of the modifications through testing of both self-report and proxy versions in key populations, including social care users and carers. This evidence will support the finalisation of the instrument.

Several limitations should be noted. All carers and most care recipients were female, reflecting challenges in recruiting male participants. This imbalance is partly attributable to the fact that in Hungary, nursing home residents, as well as formal and informal carers, are largely female [14, 50]. We did not collect information on participants’ functional status using a formal activities of daily living (ADL) measure, which limits the description of care recipients’ level of dependency. Another limitation of this study is that not all proposed EQ-HWB-9 modifications could be tested. In some cases, differences in the source UK English version (e.g. ‘lack of control’ vs. ‘no control’) were identical in the Hungarian translation, preventing evaluation of these specific changes. Finally, respondents were primarily from Southern and Western Hungary, and their language use may differ from that of individuals in other regions or the capital. This may underline some preferences for certain modifications; for example, many respondents preferred ‘getting around inside and outside’ rather than using ‘or’ as a conjunction, as it sounded more natural to them. Such preferences may reflect regional variations in language use.

## Conclusions

In conclusion, this study established the content validity of the modified EQ-HWB-9 self-complete and proxy versions in Hungarian social care users and older adult-carer dyads. The relevance of the instrument for the social care context was confirmed, as was the comprehensibility of the questionnaire. Evidence on the proposed modifications was collected systematically, contributing to the international evidence base compiled across approximately ten countries, which will inform decisions to finalise the EQ-HWB-9.

## Supplementary Information

Below is the link to the electronic supplementary material.

Supplementary Material 1

## Data Availability

The qualitative generated and/or analysed during the current study are not publicly available.

## Abbreviations

COREQ: Consolidated criteria for reporting qualitative research
COSMIN: Consensus-based Standards for the selection of health Measurement Instruments
CA: Carer
CR: Care recipient
EQ-HWB-9: EuroQol Health and Wellbeing 9
VAS: Visual Analogue Scale
VMC: Version Management Committee
