# Supplementary material for: Qualitative testing of EQ-HWB-9 self-complete and proxy versions in older adult-carer dyads in Hungary
Source: Health Qual Life Outcomes. 2026 Aug 11;24:146. doi: 10.1186/s12955-026-02602-z (PMC13746635; doi:10.1186/s12955-026-02602-z)
Supplement: Supplementary file 1 [file 12955_2026_2602_MOESM1_ESM.docx]

**Appendices**

**Appendix 1 EQ-HWB-9 proposed modifications for testing**

| **Instructions for completion** | **Amend (Y/N)** | **Proposed modifications** |
| --- | --- | --- |
| These questions are about the last 7 days. | Y | Propose change to ‘These questions are about your health and wellbeing in the last 7 days.’ to inform respondents about the scope of the forthcoming questions. |
| Please answer all questions. There are no wrong or right answers. | Y | Propose removal of the statement to simplify. |
| Please select one response for each question. | Y | Propose change from **‘select’ to ‘choose’** to align self-report version with interviewer administered version. |
| **EQ-HWB-9 items (Response level D/F/S)** | **Amend (Y/N)** | **Proposed modifications** |
| *In the last 7 days:* |  |  |
| How much difficulty did you have getting around inside and outside? (using e.g. a walking stick or wheelchair if you normally use them) (D) | Y | 1. Propose **change of order** with the question about ‘day-to-day activities’ appearing first to improve acceptability for carers.  2. Propose **removing the stem** (*how much difficulty did you have:”)* as a common statement above the item to shorten and simplify the item.  3. Propose change from ‘**inside and outside’ to ‘inside or outside’** to allow reporting of problems in either domain. |
| How much difficulty did you have doing day-to-day activities? (e.g. working, shopping, housework) (D) | Y | 1. Propose **change of order** as noted above.  2. Propose **removing the stem** as noted above. |
| *In the last 7 days, did you:* |  |  |
| feel exhausted? (F) | N | No change proposed other than to the response options. |
| feel lonely? (F) | N | No change proposed other than to the response options. |
| have trouble concentrating or thinking clearly? (F) | Y | Propose change of wording from **‘trouble’ to ‘difficulty’** to improve translatability. |
| feel anxious? (F) | N | No change proposed other than to the response options. |
| feel sad or depressed? (F) | N | No change proposed other than to the response options. |
| feel you had no control over your day-to-day life? (e.g. had no choice to do things or have things done for you as you liked and when you wanted) (F) | Y | Propose change of wording of the item and the explanation in brackets to ‘feel you **lacked control** over your day-to-day life? (**e.g. you had no choice over what you did or how things were done for you)’** to reduce ambiguity. |
| Please select **one** response to describe how much physical pain you had in the last 7 days. Did you have*:*  no / mild / moderate / severe / very severe physical pain? (S) | N | No change proposed other than changing **‘select’ to ‘choose’.** |
| **Response levels** | **Amend (Y/N)** | **Proposed modifications** |
| Difficulty (D): no / slight / some / a lot of difficulty / unable | Y | Change ‘**some’ to ‘moderate’** to reduce ambiguity. |
| Frequency (F): none of the time / only occasionally / sometimes / often / most or all of the time | Y | Change ‘**only occasionally’ to ‘a little of the time"** to reduce ambiguity. |
| Severity (S): no / mild / moderate / severe / very severe | N | No change proposed. |

***© EuroQol Research Foundation. EQ-HWB^TM^ is a trade mark of the EuroQol Research Foundation. This is a modified EQ-HWB. Reproduced by permission of EuroQol Research Foundation. Reproduction of this version is not allowed. For reproduction, use or modification of the EQ-HWB (any version), please register your study by using the online EQ registration page:*** [***www.euroqol.org***](http://www.euroqol.org)
